# Medical Student Preparedness to Counsel Parents on Childhood Vaccines and Address Vaccine Hesitancy: A Cross-Sectional Survey

**DOI:** 10.1177/23821205261463587

**Published:** 2026-06-22

**Authors:** Christina Hermann, Justen Aprile

**Affiliations:** 1 12310 Penn State College of Medicine; 2 Penn State Health Children’s Hospital

**Keywords:** medical education, pediatrics, health communication, immunization, vaccine

## Abstract

**Objective:**

Vaccine hesitancy is a significant public health concern. This study evaluated medical students’ confidence in counseling parents on childhood vaccines before and after completing the pediatrics clerkship.

**Methods:**

A cross-sectional survey of 59 Penn State College of Medicine students compared pre- and post-clerkship confidence in vaccine communication. Quantitative data were descriptively analyzed; qualitative responses explored hesitancy contributors and training suggestions. The reporting of his study followed the STROBE (Strengthening The Reporting of Observational Studies in Epidemiology) guideline for cross-sectional studies.

**Results:**

Post-clerkship students reported higher confidence in discussing vaccines (79% vs. 33%), explaining schedules (62% vs. 20%), addressing risks/benefits (7% 2vs. 43%), and countering misinformation (59% vs. 37%) compared with pre-clerkship students. Several of these differences reached statistical significance. Comfort discussing vaccine safety remained low (∼44%). Students identified misinformation, politics, and social media as key hesitancy drivers and recommended standardized patient practice and formal teaching to improve preparedness.

**Conclusion:**

Pediatrics clerkship clinical exposure boosts medical student confidence in vaccine counseling. Targeted skills training is needed to address safety discussions and parental persuasion challenges.

## Purpose/Research Question

This study examined the level of preparedness and confidence among medical students in counseling parents about childhood vaccines and addressing vaccine hesitancy. Specifically, we investigated how confidence differs before and after completion of the pediatrics clerkship.

We hypothesized that students who completed the pediatrics clerkship would report higher levels of confidence and preparedness compared with students who had not yet begun the clerkship.

## Introduction

Vaccine hesitancy is a growing public health concern, contributing to decreased vaccination rates and increased outbreaks of vaccine-preventable diseases.^1^ Although childhood vaccination remains one of the most effective strategies for reducing morbidity and mortality, parental concerns continue to challenge healthcare providers’ efforts to communicate the importance and safety of vaccines.^2,3^ Physicians play a critical role in influencing parental decisions about childhood vaccinations through effective communication.^4,5^

Despite this, little is known about medical students’ readiness to engage in these conversations or how their confidence evolves throughout their training. While previous research has evaluated confidence and communication skills among healthcare providers,^6,7^ limited data exist on medical students’ preparedness or the impact of clinical experiences on preparedness. Understanding these factors is essential for identifying gaps in medical student training and strengthening educational strategies to promote effective communication.

This study aims to understand this gap by assessing medical students’ self-reported confidence, attitudes, and perceived preparedness in counseling parents about childhood vaccines and responding to vaccine-hesitant parents. By comparing responses between pre-pediatrics clerkship and post-pediatrics clerkship students, we seek to evaluate how clinical exposure, and formal education may influence preparedness. Insights from this study can inform curriculum development and enhance training for future physicians as they confront increasing rates of vaccine hesitancy.

The primary objective of this study was to compare self-reported confidence in vaccine counseling between students who had and had not completed the pediatrics clerkship. Secondary objectives included exploring attitudes toward vaccine hesitancy, identifying perceived contributors to vaccine hesitancy, and identifying educational strategies that students believed would improve preparedness for these conversations.

## Methods

### Study Design and Population

This cross-sectional survey study was conducted to assess medical students’ confidence, preparedness, and attitudes regarding counseling parents on childhood vaccines and addressing vaccine hesitancy. Participants included all medical students (MS1-MS4) enrolled at Penn State College of Medicine.

Inclusion criteria included current enrollment as a medical student at Penn State College of Medicine and voluntary completion of the survey. Exclusion criteria included incomplete surveys or failure to indicate pediatrics clerkship status.

For analysis, students were categorized based on their completion of the pediatrics clerkship, which did not strictly align with class year, as many third-year students had not completed the pediatrics clerkship at the time of survey administration. This approach allowed for comparison between pre-clerkship students and post-clerkship students, with particular focus on the impact of the pediatrics clerkship on students’ self-reported confidence and preparedness.

The pediatrics clerkship at Penn State College of Medicine is a required four-week rotation in which students care for children from birth to 18 years of age across inpatient, outpatient, and newborn nursery settings. During the clerkship, students learn pediatric history-taking and physical examination skills, provide anticipatory guidance and preventive counseling, and manage a wide range of conditions, including opportunities to participate in discussions with families about childhood immunizations.

During clinical experiences, students observed and participated in real-time discussions regarding routine immunizations, vaccine safety, common misconceptions, and parental vaccine concerns under faculty and resident supervision. While there was no standardized vaccine communication curriculum or formal standardized patient exercise specifically dedicated to vaccine hesitancy during the clerkship, students were exposed to vaccine counseling through clinical encounters in outpatient pediatric settings.

### Data Collection and Survey Instrument

Data were collected through an anonymous online survey administered via REDCap and distributed to all enrolled medical students through institutional email lists and student groups. The survey took approximately 5-7 minutes to complete and included both multiple choice and Likert-scale questions assessing:

- Comfort discussing routine childhood vaccines
- Preparedness to explain the childhood vaccine schedule
- Ability to communicate the risks and benefits of vaccination
- Confidence in explaining vaccine safety and mechanism of action
- Comfort addressing common vaccine misinformation
- Perceptions of how well medical school training prepared them for vaccine hesitancy discussions
- Attitudes toward the importance of vaccine counseling in all specialties
- Emotional responses to parental vaccine refusal

Demographic variables included year in medical school, completion of pediatrics clerkship, exposure to formal vaccine education, and prior experience with vaccine-hesitant families. Optional open-ended questions allowed participants to describe clinical encounters and suggest improvements to vaccine communication training.

Preparedness was defined as students’ self-reported perception of their ability to effectively explain vaccines, address misinformation, discuss risks and benefits, and navigate vaccine-hesitant conversations. Comfort, confidence, and preparedness were measured using Likert-scale responses.

The survey questions were developed based on the study objectives, prior literature on vaccine communication, and review by faculty with experience in pediatrics and medical education to support content validity. The survey was pilot reviewed for clarity and relevance prior to distribution. However, a formal psychometric validation process was not performed. The survey questions are included in Supplementary File 1.

### Sample Size

The target sample size was approximately 100 students, representing roughly 20% of the total medical student body at Penn State College of Medicine.

A formal priori power calculation was not performed. The target sample size of approximately 100 students was selected to provide adequate representation across training years and allow comparison between pre- and post-clerkship groups. However, only 59 students completed the survey, which may have limited statistical power to detect smaller differences between groups. This is addressed further in the limitations section.

### Data Analysis

Quantitative data were analyzed using descriptive statistics, including frequencies, percentages, and means.

Comparisons between pre-clerkship and post-clerkship students were conducted using chi-square or fisher exact tests, as appropriate for small cell sizes. A p-value < 0.05 was considered statistically significant. Given the exploratory nature of this study and the modest sample size, statistical results were interpreted cautiously.

For open ended questions, qualitative content analysis identified common themes regarding barriers and educational needs related to vaccine counseling.

Responses to open-ended questions were not mutually exclusive, and participants could identify multiple themes within a single response.

The reporting of this study confirms to the STROBE (Strengthening the Reporting of Observational Studies in Epidemiology) statement.^8^ The completed STROBE checklist is provided as Supplementary File 2.

### Ethical Considerations

This study was conducted at Penn State College of Medicine and received approval from the Institutional Review Board (STUDY00027866). Participation was voluntary and anonymous, with informed consent obtained electronically at the survey’s start. No identifying information was collected.

## Results

### Participant Characteristics

A total of 59 medical students completed the survey. Of these, 30/59 (50.8%) had not completed the pediatrics clerkship (pre-clerkship group), while 29/59 (49.2%) had completed the clerkship (post-clerkship group).

There was no significant difference between the two groups in the proportion of students who reported encountering vaccine-hesitant families in clinical settings.

For all analyses, students were grouped based on completion of the pediatrics clerkship completion rather than class year because approximately half of the MS3 cohort had completed the clerkship and half had not at the time of survey distribution.

### Comfort Level and Confidence Discussing Vaccines

Table 1 summarizes the frequencies and percentages of pre- and post-clerkship students who agreed with statements related to vaccine communication skills and preparedness.

**Table 1.** Frequency and Percentage of Medical Students Agreeing With Vaccine Communication Statements, by Pediatrics Clerkship Completion

Statement/Measure	Pre-clerkshipagree n/N (%)	Post-clerkshipagree n/N (%)	P value
Comfort Discussing Vaccines	10/30 (33.4%)	23/29 (79.3%)	*P* < 0.01
Comfort Explaining Vaccine Schedule	6/30 (20%)	18/29 (62.1%)	*P* < 0.01
Comfort Discussing Risks/Benefits	13/30 (43.3%)	21/29 (72.4%)	*P* = 0.03
Confidence Describing Vaccine Protection	24/30 (80%)	27/29 (93.1%)	*P* = 0.17
Comfort Discussing Vaccine Safety	13/30 (43.4%)	13/29 (44.8%)	*P* = 0.91
Preparedness to Explain How Vaccines Work	21/30 (70%)	20/29 (69%)	*P* = 0.93
Comfort Addressing Misinformation	11/30 (36.7%)	17/29 (58.6%)	*P* = 0.09

Post-clerkship students reported significantly greater comfort discussing vaccines overall, explaining the childhood vaccine schedule, and discussing vaccine risks and benefits compared with pre-clerkship students. Confidence describing vaccine protection and preparedness explaining how vaccines work remained high in both groups. However(Table 2), comfort discussing vaccine safety remained similarly limited across both groups.

**Table 2.** Frequency and Percentage of Medical Students Agreeing With Attitudinal Statements, by Pediatrics Clerkship Completion

Attitudinal measure	Pre-clerkshipagree n/N (%)	Post-clerkshipagree n/N (%)
Vaccine discussions are important for all physicians	30/30 (100%)	28/29 (96.5%)
Medical school prepared students to address vaccine hesitancy	3/30 (10.0%)	12/29 (41.4%)
Belief that parents can be persuaded	13/30 (43.3%)	9/29 (31.0%)
Frustration with vaccine refusal	19/30 (63.4%)	20/29 (69.0%)
Perception that vaccine hesitancy is increasing	23/30 (76.7%)	26/29 (89.6%)

### Additional Attitudinal Findings

Although post-clerkship students reported greater confidence in vaccine counseling skills, they were less likely to believe that vaccine-hesitant parents could be persuaded. Frustration with vaccine refusal was high in both groups.

## Open-Ended Responses

### Perceived Contributors to Vaccine Hesitancy

Forty-seven students responded to the optional question regarding contributors to vaccine hesitancy. Of the 47 respondents(Table 3), 24 were pre-clerkship students and 23 were post-clerkship students.

**Table 3.** Medical Students’ Perceived Contributors to Vaccine Hesitancy

Contributor to vaccine hesitancy	Number of responses	Percentage (%)
Misinformation	35	74.5
Politics/Government Influence	12	25.5
Social Media/News Media	11	23.4
Mistrust or Fear of Medical System	9	19.1
Religion/Cultural Values	5	10.6
COVID-19 Related Concerns	3	6.4

Misinformation was the most commonly identified contributor to vaccine hesitancy, followed by political influences and social media.

### What Would Improve Preparedness to Address Vaccine Hesitancy

Forty-one students responded to the optional question regarding strategies that would improve preparedness for vaccine counseling. Of the 41 respondents, 20 were pre-clerkship students and 21 were post-clerkship students(Table 4). Responses were not mutually exclusive.

**Table 4.** Medical Students’ Suggested Strategies to Improve Preparedness for Counseling Vaccine-Hesitant Parents

Suggested improvement	Number of responses	Percentage (%)
Practice with Standardized Patients	17	41.5
Clear Talking Points or Rebuttals	16	39.0
Formal Teaching	10	24.4
Integrating Cultural Considerations	2	4.9
Observing Clinician Role Models	2	4.9
Providing Handouts	1	2.4
Increased Governmental Support	1	2.4
Allowing More Time for Discussions	1	2.4

Students most frequently recommended increased opportunities for standardized patient encounters, structured talking points for vaccine counseling, and additional formal teaching on vaccine hesitancy communication.

## Discussion

This study examined medical students’ self-reported comfort and preparedness in counseling parents about childhood vaccines and addressing vaccine hesitancy, with a particular focus on the impact of the pediatrics clerkship. Consistent with our hypothesis, students who had completed the pediatrics clerkship reported higher confidence across multiple domains, including overall comfort discussing vaccines, explaining the childhood vaccine schedule, discussing risks and benefits, and addressing misinformation. These findings suggest that clinical exposure and early opportunities to practice communication play an important role in developing students’ vaccine counseling skills.

Confidence in explaining how vaccines work and how they protect communities was high in both pre- and post-clerkship groups, indicating that foundational immunology knowledge is generally well understood regardless of training stage, as all students surveyed had already completed their pre-clinical foundational immunology course. However, comfort discussing vaccine safety remained relatively low across both groups. This persistent discomfort may represent more than an isolated skill gap. Parents who express vaccine hesitancy commonly focus on safety concerns, adverse effects, and risk-benefit questions. Students who feel less comfortable navigating these discussions may experience greater frustration during clinical encounters and may develop lower expectations regarding their ability to persuade vaccine-hesitant parents.

The observed increases in confidence are consistent with previous research demonstrating that direct patient encounters enhance trainees’ (i.e.,residents, fellows) communication skills and their ability to navigate challenging conversations.^9^ Prior studies also suggest that supervised practice and targeted educational interventions increase clinicians’ comfort in addressing vaccine hesitancy.^10,11^ Our findings extend these observations by demonstrating similar trends at the medical student level, suggesting that even brief exposure during a pediatrics rotation can positively influence perceived preparedness for vaccine counseling.

Despite these gains, students in both groups expressed mixed perceptions regarding their ability to persuade vaccine-hesitant parents. Notably, post-clerkship students were more likely to disagree that parents can be persuaded, potentially reflecting increased awareness of the complexities and emotional challenges surrounding vaccine refusal. Rather than representing purely negative findings, these attitudinal shifts may reflect the development of greater clinical realism. Students who enter clerkships with high expectations about their ability to change parental beliefs may leave with a more nuanced understanding of the social, emotional, cultural, and political factors that shape vaccine decision-making. Such realism may actually be important for long-term professional development, as effective vaccine counseling often requires repeated conversations, trust-building, empathy, and motivational interviewing rather than immediate persuasion.

Open-ended responses further revealed students’ perceptions of contributors to vaccine hesitancy, with misinformation, political influences, and social media being the most frequently cited factors. These themes are consistent with existing literature describing the impact of misinformation and politicization of public health on parental decision-making.^11^ Students also suggested strategies to improve preparedness, including practice with standardized patients, clear talking points or rebuttals, and formal curricular instruction.

These findings suggest that medical education should not only focus on increasing factual knowledge about vaccines but also prepare students for the emotional and interpersonal complexities of vaccine-hesitant encounters. Specific educational strategies may include standardized patient scenarios focused on vaccine safety concerns, communication workshops emphasizing motivational interviewing and empathetic listening, faculty debriefing after difficult encounters, and longitudinal opportunities to observe experienced clinicians navigate challenging vaccine discussions. Such approaches may help students maintain constructive engagement and empathy while developing realistic expectations about the limits of persuasion in individual clinical encounters.

This study has several strengths, including participation from students across multiple training years and the use of both quantitative and qualitative data to provide a broader understanding of preparedness and attitudes toward vaccine counseling. Comparing students before and after completion of the pediatrics clerkship allowed for direct evaluation of the impact of clinical experience on confidence.

### Limitations

However, the study has several limitations. The final sample size of 59 participants was smaller than the initial target sample and may have limited statistical power to detect small between-group differences. Therefore, results should be interpreted as exploratory and hypothesis-driven rather than definitive. Additionally, the study relief on self-reported confidence rather than observed communication performance, and the survey instrument did not undergo formal psychometric validation. Because participation was voluntary and conducted through an online survey, selection bias, accessibility bias, and social desirability bias are also possible.

Despite these limitations, the findings highlight important gaps in medical student training related to vaccine communication. Integrating targeted educational strategies, such as structured simulation encounters, skills-based workshops, and clearer messaging frameworks, may better prepare future physicians to navigate conversations with vaccine-hesitant parents. As vaccine hesitancy continues to rise nationally, equipping trainees with robust communication skills is increasingly essential.

## Conclusion

Medical students’ confidence and preparedness in discussing childhood vaccines improve following completion of the pediatrics clerkship, particularly in areas such as explaining vaccine schedules, discussing risks and benefits, and addressing misinformation.

However, improved confidence did not necessarily translate into greater optimism about persuading vaccine-hesitant parents. Post-clerkship students may develop a more realistic understanding of the challenges involved in vaccine counseling, particularly when safety concerns, misinformation, and emotionally charged conversations are involved. These findings suggest that effective training should prepare students not only to communicate factual information, but also to manage frustration, build trust, and remain empathetic during difficult discussions.

Foundational knowledge about vaccine mechanisms and community protection is high even before clinical exposure, but comfort discussing vaccine safety remains limited. These findings support the integration of targeted, skills-based communication training, including standardized patient encounters, motivational interviewing practice, and structured vaccine counseling exercises, into medical school curricula.

## Supplemental Material

Supplemental Material - Medical Student Preparedness to Counsel Parents on Childhood Vaccines and Address Vaccine Hesitancy: A Cross-Sectional Survey Supplemental Material for Medical Student Preparedness to Counsel Parents on Childhood Vaccines and Address Vaccine Hesitancy: A Cross-Sectional Survey by Christina Hermann, BS, Justen Aprile, MD in Journal of Medical Education and Curricular Development.
