# Supplementary material for: Medical Student Preparedness to Counsel Parents on Childhood Vaccines and Address Vaccine Hesitancy: A Cross-Sectional Survey
Source: J Med Educ Curric Dev. 2026 Jun 22;13:23821205261463587. doi: 10.1177/23821205261463587 (PMC13305800; doi:10.1177/23821205261463587)
Supplement: Supplemental Material - Medical Student Preparedness to Counsel Parents on Childhood Vaccines and Address Vaccine Hesitancy: A Cross-Sectional Survey [file sj-pdf-1-mde-10.1177_23821205261463587.pdf]

Med Student Survey

This anonymous survey is being conducted to assess medical students' knowledge, confidence, and attitudes toward childhood vaccinations, including their preparedness to counsel vaccine-hesitant families. Your participation is voluntary and confidential. The survey takes approximately 5-7 minutes to complete.

By continuing, you consent to participate.

Thank you!

What year are you in medical school?	◯ MS1<br>◯ MS2<br>◯ MS3<br>◯ MS4
Have you completed your pediatrics clerkship?	◯ Yes<br>◯ No
What specialty are you planning to apply into? (optional)	__________________________________
Have you received formal teaching on childhood vaccines?	◯ Yes<br>◯ No<br>◯ Not sure
Have you encountered vaccine-hesitant parents during your clinical training?	◯ Yes<br>◯ No<br>◯ Not sure
Please indicate your level of agreement with the following statement.<br><br>I feel comfortable discussing routine childhood vaccines with parents.	◯ Strongly agree<br>◯ Agree<br>◯ Neutral<br>◯ Disagree<br>◯ Strongly disagree
Please indicate your level of agreement with the following statement.<br><br>I feel prepared to explain the vaccine schedule to families.	◯ Strongly agree<br>◯ Agree<br>◯ Neutral<br>◯ Disagree<br>◯ Strongly disagree
Please indicate your level of agreement with the following statement.<br><br>I am comfortable discussing the risks and benefits of vaccines in a balanced way.	◯ Strongly agree<br>◯ Agree<br>◯ Neutral<br>◯ Disagree<br>◯ Strongly disagree
Please indicate your level of agreement with the following statement.<br><br>I feel confident explaining how vaccines protect children and communities.	◯ Strongly agree<br>◯ Agree<br>◯ Neutral<br>◯ Disagree<br>◯ Strongly disagree
Please indicate your level of agreement with the following statement.<br><br>I feel confident discussing vaccine safety with hesitant families.	◯ Strongly agree<br>◯ Agree<br>◯ Neutral<br>◯ Disagree<br>◯ Strongly disagree

Please indicate your level of agreement with the following statement.<br><br>I feel prepared to explain how vaccines work in a clear and non-judgmental way.	◯ Strongly agree<br>◯ Agree<br>◯ Neutral<br>◯ Disagree<br>◯ Strongly disagree
Please indicate your level of agreement with the following statement.<br><br>I feel comfortable addressing misinformation (ex: autism, toxins, etc)	◯ Strongly agree<br>◯ Agree<br>◯ Neutral<br>◯ Disagree<br>◯ Strongly disagree
Please indicate your level of agreement with the following statement.<br><br>Addressing vaccine hesitancy is an important skill for all physicians, regardless of specialty.	◯ Strongly agree<br>◯ Agree<br>◯ Neutral<br>◯ Disagree<br>◯ Strongly disagree
Please indicate your level of agreement with the following statement.<br><br>Medical school has adequately prepared me to address vaccine hesitancy.	◯ Strongly agree<br>◯ Agree<br>◯ Neutral<br>◯ Disagree<br>◯ Strongly disagree
Please indicate your level of agreement with the following statement.<br><br>I believe most parents who are hesitant about vaccines can be persuaded with<br>evidence and empathy.	◯ Strongly agree<br>◯ Agree<br>◯ Neutral<br>◯ Disagree<br>◯ Strongly disagree
Please indicate your level of agreement with the following statement.<br><br>I feel frustrated when families refuse vaccines despite my efforts.	◯ Strongly agree<br>◯ Agree<br>◯ Neutral<br>◯ Disagree<br>◯ Strongly disagree
Please indicate your level of agreement with the following statement.<br><br>Vaccine hesitancy is becoming more common in clinical settings.	◯ Strongly agree<br>◯ Agree<br>◯ Neutral<br>◯ Disagree<br>◯ Strongly disagree
What do you think contributes to vaccine hesitancy?	__________________________________
What would help you feel more confident when counseling vaccine-hesitant<br>families?	__________________________________
Do you have any experiences you'd like to share about encountering vaccine<br>hesitancy during your training?	__________________________________
